# English Validation of the Parental Socialization Scale—ESPA29

**DOI:** 10.3389/fpsyg.2017.00865

**Published:** 2017-05-29

**Authors:** Isabel Martínez, Edie Cruise, Óscar F. García, Sergio Murgui

**Affiliations:** ^1^Psychology Department, University of Castilla-La ManchaCuenca, Spain; ^2^Department of Developmental and Educational Psychology, University of ValenciaValencia, Spain; ^3^Social Psychology Department, University of ValenciaValencia, Spain

**Keywords:** parenting practices, socialization, Parental Socialization Scale, ESPA29, validation

## Abstract

Parenting styles have traditionally been studied following the classical two-dimensional orthogonal model of parental socialization. The Parental Socialization Scale ESPA29 is used to measure the four styles of parental socialization through the acceptance/involvement and strictness/imposition dimensions. The ESPA29 scale is a developmentally appropriate measure of parenting styles, which has been validated in several languages including Spanish, Italian, and Brazilian Portuguese. In this study, the English translation of the ESPA29 was evaluated. The objective of the work is to test the ESPA29’s structure of parenting practices with a United States sample measuring parenting practices using exploratory factor analysis (EFA) and confirmatory factor analysis (CFA). The scores of fathers’ and mothers’ behavioral practices toward their children were obtained for a sample of 911 United States adolescents between 14 and 18 years of age. First, the total sample was split and a principal components analysis with varimax rotation was carried out with one of the two halves. EFA showed a two-factor structure fully congruent with the theoretical model for mothers’ and fathers’ scores. Next, a CFA was calculated on the second half by using the factor structure obtained in the previous EFA. The CFA replicated the two-factor structure with appropriate fit index. The seven parenting practices that were measured loaded appropriately on the acceptance/involvement and strictness/imposition dimensions. Then, the multigroup analysis between girls and boys showed equal loading in the factors and equal covariation between the acceptance/involvement and the strictness/imposition dimensions. Additionally, the two dimensions of the ESPA29 scale were related to self-esteem in order to obtain an external validity index. The findings confirm the invariant structure of the ESPA29 was in the United States and their equivalence in both fathers’ and mothers’ scores. These findings validate the instrument and confirm its applicability in cross-cultural research on parenting practices and child adjustment.

## Introduction

Styles of family socialization and the way these styles are conceptualized and measured are key in parenting research (Maccoby and Martin, 1983; Gray and Steinberg, 1999). Styles allow for a great part of the relationship established between parents and children to be classified (Darling and Steinberg, 1993). Parenting styles also enable parental behavior to be related to different child adjustment variables with greater clarity and consistency than considering isolated parenting practices (Symonds, 1939). The relations between parenting styles and child adjustment have traditionally been studied following the classical two-dimensional orthogonal model of parental socialization. Since the work of Maccoby and Martin (1983), these two parental socialization dimensions have frequently been denominated as demandingness and responsiveness (Steinberg, 2005). Earlier scholars have used other labels such as acceptance (Symonds, 1939), assurance (Baldwin, 1955), warmth (Sears et al., 1957; Becker, 1964), or love (Schaefer, 1959), which have similar meanings to responsiveness. Labels such as domination, hostility, inflexibility, control, firmness, or restriction were used in earlier research with similar meanings to demandingness (Symonds, 1939; Sears et al., 1957; Schaefer, 1959; Becker, 1964). The demandingness dimension refers to the extent to which parents use control, and supervision, make maturity demands, and maintain an assertive position of authority with their children. The responsiveness dimension represents the degree to which parents show their child warmth and acceptance, give them support, and communicate by reasoning with them (Becker, 1964; Martínez and García, 2008). Based on these two dimensions, four parental socialization styles are identified: authoritative style—characterized by the use of high demandingness and high responsiveness; neglectful style—characterized by low demandingness and low responsiveness; indulgent style—low demandingness and high responsiveness; and authoritarian style—high demandingness and low responsiveness (Lamborn et al., 1991).

Among the scales used to measure the four styles of parental socialization through two dimensions is the authoritative parenting measure (Lamborn et al., 1991; Steinberg et al., 1992). In this scale, the four parenting typologies are created on the basis of adolescents’ scores on two of the dimensions measured by this instrument: the acceptance/involvement and strictness/supervision dimensions (e.g., Lamborn et al., 1991; Chao, 2001). The acceptance/involvement scale looks at the degree to which adolescents perceive their parents as responsive, caring, and involved. The strictness/supervision scale measures the degree to which parents regulate and monitor adolescent behavior and whereabouts (Lamborn et al., 1991; Steinberg et al., 1992). Other commonly used scales that measure the four parenting styles though two dimensions are the Warmth/Affection Scale (WAS; Rohner et al., 1978; Rohner, 2005) and the Parental Control Scale (PCS; Rohner, 1989; Rohner and Khaleque, 2003). These two scales have been used jointly to create the four parenting styles typology (Kim and Rohner, 2002). The WAS measures the extent to which adolescents perceive their parents as loving, responsive, and involved, whereas the PCS assesses the extent to which an adolescent perceives strict parental control in their parents’ behavior. Both scales have been used across culturally distinct populations (Rohner and Khaleque, 2003). However, those instruments do not contemplate the differentiation between practices and styles of socialization and do not use a contextual or situational perspective to measure parenting behavior (Darling and Steinberg, 1993; Smetana et al., 2006).

Additionally, in the research of parenting behavior, other instruments have been used to assess three parenting styles of socialization, following the pioneering work of Baumrind (1967, 1972, 1983), as in, for example, the widely used Parenting Styles and Dimensions Questionnaire (PSDQ), developed by Robinson et al. (1995), or the Parental Authority Questionnaire (Buri, 1991), both instruments have been developed for the purpose of measuring Baumrind’s (1971) permissive, authoritarian, and authoritative parental prototypes. However, the Baumrind’s initial tripartite model does not contemplate the differentiation between neglectful and indulgent parenting, as Lamborn et al. (1991) observed “most discussions and empirical tests of Baumrind’s model… ignore variations in warmth among families characterized by low levels of control, grouping these families together into a single category labeled ‘permissive”’ (p. 1050). Contrastingly, the four-typology or quadripartite model stressed the need to consider the combination of the two parenting dimensions in the analysis of its relationships with youth outcomes (Lamborn et al., 1991).

The Parental Socialization Scale ESPA29 (Musitu and García, 2001) is a four-typology parenting measure that was specifically developed to measure the four parental socialization styles using a contextual (Darling and Steinberg, 1993) and situational (Smetana, 1995) perspective. This instrument specifically evaluates parental behaviors in concrete situations representative of family life, asking the offspring about their parents’ behavior in specific situations that are likely to occur in Western culture. Additionally, the instrument purposely contemplates the differentiation between parenting practices and styles (Darling and Steinberg, 1993; Kerr and Stattin, 2000). First, the scale measures the use made by parents of seven different practices of socialization: warmth, indifference, reasoning, detachment, verbal scolding, physical punishment, and revoking privileges. These practices form two socialization dimensions—acceptance/involvement and strictness/imposition—which have equivalent meanings to the classical dimensions of responsiveness and demandingness (Lamborn et al., 1991). Finally, the four parenting styles—authoritative, authoritarian, indulgent, and neglectful—are created from the parents’ scores in the acceptance/involvement and strictness/imposition dimensions.

**FIGURE 1:**
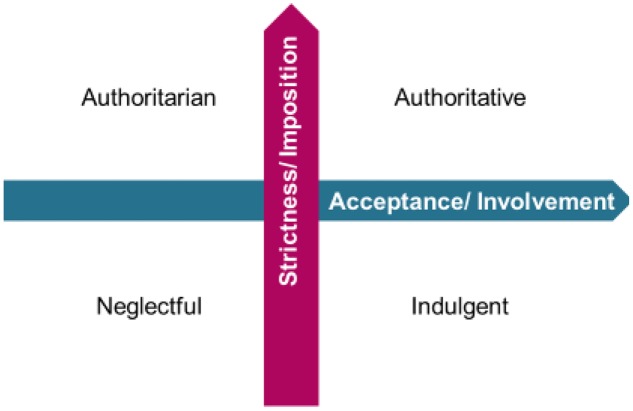
Bi-dimensional model of parental socialization.

**FIGURE 2:**
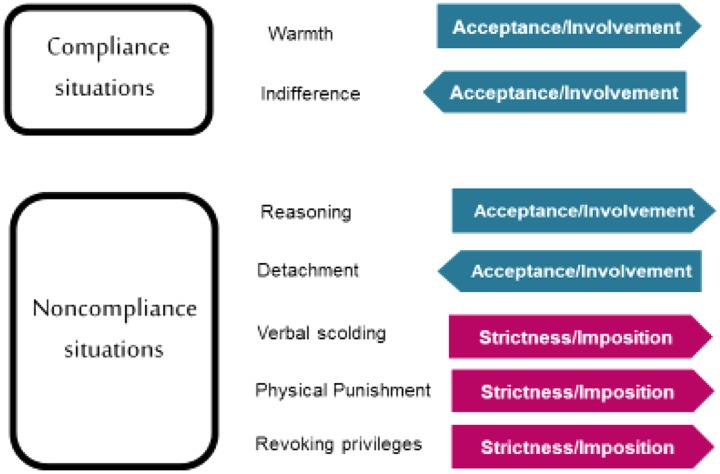
ESPA29 parenting practices and dimensions of socialization.

In the ESPA29, parenting practices are organized on the two-dimensional model (**Figure 1**) according to a theoretical structure that distinguishes between situations of adolescents’ compliance and non-compliance with family norms (**Figure 2**). The practices of verbal scolding, physical punishment, and revoking privileges are measured in situations of non-compliance. These three practices are positively related to the strictness/imposition dimension (**Figure 2**) and are intended to correct undisciplined behavior by imposing restrictions and limits on the child’s or adolescent’s conduct. The desired outcome in the child or adolescent, as the process of socialization implies, is to assist the child or adolescent in developing the ability to suppress attractive yet prohibited behaviors and adopt others that are more socially acceptable (Mischel and Mischel, 1976). Additionally, the practices of reasoning and detachment are also measured in situations of non-compliance. These two practices are negatively related to each other and are placed on the dimension of acceptance/involvement (**Figure 2**). The practice of reasoning is intended to correct undisciplined behavior, as are the practices of the strictness/imposition dimension. Finally, in situations of compliance the practices of warmth and indifference are measured (**Figure 2**), which are also located on the acceptance/involvement dimension. The two practices are negatively related to each other and allow for the correct behavior of the child to either be recognized or ignored (Baumrind, 1983; Grusec, 2012). The recognition of the child’s adjusted conduct through warmth relates positively to the use of reasoning practices given that both parenting practices—warmth and reasoning—require a long-term, optimal parent–child relationship in order to take place (Musitu and García, 2001).

The original version of the Parental Socialization Scale ESPA29 was first developed and validated in Spain (Musitu and García, 2001). This instrument was designed to assess parenting styles through self-reports of children and adolescents from 10 to 18 years old, but it has been mainly used with older adolescents (e.g., Martínez and García, 2008; Martínez et al., 2013). Subsequently, it has been validated for use in a number of other languages, including the Basque language (López-Jáuregui and Oliden, 2009), Italian (Marchetti, 1997), and Portuguese (Martínez et al., 2011; Martínez I. et al., 2012). All of these validation studies confirm the theoretical factor structure of the ESPA29. In addition, recently the concurrent validity of the ESPA29 has been tested satisfactorily in two different Spanish samples (García and Gracia, 2014; García et al., 2015). Although exploratory factor analyses (EFAs) have consistently identified the theoretical factor structure of the ESPA29, previous studies that have attempted to apply the confirmatory factor analysis (CFA) have failed to provide support for the ESPA29 structure (see López-Jáuregui and Oliden, 2009). In this study, we have applied robust CFA in contrast to previous studies that only applied Procrustes Rotations (e.g., Hayton et al., 2004; Marsh et al., 2010; Veronese and Pepe, 2016).

Additionally, the ESPA29 has been widely used in Spain (Martínez and García, 2007; García and Gracia, 2009, 2010; Martínez et al., 2013; Fuentes et al., 2015a,b,c) and also in Portugal (Rodrigues et al., 2013) in order to study the relations between socialization styles and different adolescent adjustment variables. These studies have also measured parenting styles congruently and point out the importance of the practices of the acceptance/involvement dimension in adolescent adjustment. For example, it has been found that in Spain, adolescents raised with an indulgent socialization style show the highest levels of self-esteem, similar or superior to those of adolescents raised with an authoritative style (Musitu and García, 2004; García et al., 2015). Similar results have been found with other adjustment criteria, such as value internalization (Martínez and García, 2007), personal competence, and problem behavior (García and Gracia, 2009, 2010; Martínez et al., 2013). Furthermore, the ESPA29 scale has been used to relate parenting to adolescent adjustment in Brazil (Martínez et al., 2007, 2014) and Peru (Albertí et al., 2015). In these South American countries, the use of indulgent parenting also seems to be related to good adolescent adjustment, also similar or higher than the use of authoritative parenting. These results reveal the importance of the acceptance/involvement practices, common to both the indulgent and authoritative styles, as key in adolescent self-esteem and adjustment in general. However, the ESPA29 has not been used in English-speaking countries where most of the parenting research has been carried out.

Hence, the objective of this work is to test the ESPA29’s structure of parenting practices with a United States sample measuring the practices of fathers and mothers, and testing the gender invariance for boys and girls. The ESPA29 adapts universal parenting practices to Western culture as its basis to define the two dimensions of socialization—acceptance/involvement and strictness/imposition—. The bi-dimensional structure of the instrument has already been identified in other languages and countries for fathers’ and mothers’ practices (López-Jáuregui and Oliden, 2009; Martínez I. et al., 2012). Thus, we expect that the ESPA29’s theoretical structure will be confirmed in the United States and will be equivalent in both fathers and mothers, as well as invariant for boys and girls. Additionally, both of the ESPA29’s scale dimensions—acceptance/involvement and strictness/imposition—will be related to self-esteem, a classic criterion variable used in parenting studies (Jimenez et al., 2007; Murgui et al., 2016) in order to obtain an external validity index. According to the results in previous research (Musitu and García, 2001; Garaigordobil et al., 2015), it is expected that the use of acceptance/involvement practices will be related positively with adolescent self-esteem, whereas the use of strictness/imposition dimension will be related negatively with self-esteem.

## Materials and Methods

### Participants

The sample of the present study consisted of 911 adolescents (cases with missing values were deleted listwise). All the subjects attending public high school in middle class neighborhoods in a city of approximately 250,000 inhabitants in the Midwest of the United States. Girls made up 53.9% of the sample and boys made up the remaining 46.1%. The participants ranged in age from 14 to 18 years old. The mean age was of 16.13 (*SD* = 1.09). Each age group had the following number of participants (in parentheses): 14 (85), 15 (151), 16 (311), 17 (283), and 18 (81). Participants identified their ethnicity according to their parents’ background. They reported the ethnicity of their fathers and mothers as: European-Americans, 79.5% fathers and 82.0% mothers; Asian-Americans, 6.5% and 7.1%; African-Americans, 5.3% and 2.9%; Hispanic-Americans, 5.2% and 4.5%; Native-Americans, 3.5% and 3.6%, respectively.

### Procedure

The data was collected in five educational centers selected by simple random sampling from a complete list of centers in the region. According to Kalton (1983), when groups (i.e., educational centers) are selected randomly, the elements that make up those groups (i.e., students) will be similar to what a random system would provide. The Ethics Committee at the University of University of Castilla-La Mancha, where the research was designed, granted ethical approval for the study. Permission was first obtained to conduct this study in public high schools from the Research and Evaluation Board of the Public School Board in the city where the research took place. Then it was necessary to receive permission from the individual principals of each high school. Once the principals allowed for the study to be carried out in the schools, individual teachers had to agree to the administration of the questionnaire during their class time. Finally, permission from the students’ parents had to be granted, along with assent from the students themselves. The researchers administered the instruments to all the students who had permission to participate. The questionnaire included the ESPA29 and the AF5 scales and demographic data of the participants. It took about 20 min to complete and the gathering phase finish on January 2016. All of the questionnaires were completed anonymously.

### Instruments

#### The Parental Socialization Scale ESPA29

In this scale (Musitu and García, 2001), the youth rates the frequency with which both their father and mother (considered separately) employ different socialization practices in response to 29 situations that are representative of everyday family life. The frequency of the practices’ use is indicated on a 4-point scale in which 1 = “never,” 2 = “sometimes,” 3 = “most times,” and 4 = “always.” The 29 scenarios are divided into 13 that represent situations of obedience in which the child acts in accordance with the family norms (e.g., “If the school reports that I am well-behaved…”) and 16 that represent situations of disobedience in which the child does not conform to family norms (e.g., “If I leave home to go somewhere without asking anyone for permission…”). In the 13 situations of obedience the practices of warmth (“He/She shows warmth”) and indifference (“He/She seems indifferent) are evaluated. In the 16 situations of disobedience the practices of reasoning (“He/She talks to me”) and detachment (It’s the same to him/her”), as well as verbal scolding (“He/She scolds me”), physical punishment (“He/She hits me”) and revoking privileges (“He/She takes something away from me”) are evaluated. In total, the adolescent gives 212 responses, 106 for the father’s behavior and 106 for the mother’s behavior.

The score for the acceptance/involvement dimension is obtained by averaging the scores of the subscales of warmth, reasoning, indifference, and detachment (the subscales of indifference and detachment are inverted as they are inversely related to the dimension) for both mothers and fathers. The score for the strictness/imposition dimension is calculated by averaging the responses to the subscales of revoking privileges, verbal scolding, and physical punishment for the mother and father. Parental conduct can be classified into the four parental socialization typologies (authoritative, indulgent, authoritarian, or neglectful) by dichotomizing (Lamborn et al., 1991; Steinberg et al., 1994) the scores for the mothers’ and fathers’ behavior in the acceptance/involvement and the strictness/imposition dimensions either at the tertile (Musitu and García, 2001; Martínez and García, 2007) or at the median (Chao, 2001; Kremers et al., 2003; García and Gracia, 2009, 2010). In this way, the authoritative style is defined by high use of acceptance/involvement and strictness/imposition practices, the indulgent style by high use of acceptance/involvement and low use of strictness/imposition, the authoritarian style by low use of acceptance/involvement and high use of strictness/imposition, and finally, the neglectful style by low use of both acceptance/involvement and strictness/imposition practices.

For the translation of the ESPA29 from Spanish into English, the inverse translation method proposed by Brislin (1970) was followed in order to ensure the items were comparable to other language versions of the scale. Upon receiving permission from the authors, the original measure was translated into American English from Spanish by two native English-speaking colleagues. They performed a cross-check on item grammar, clarity, and content equivalence and the resulting items were back-translated into Spanish by an independent, bilingual researcher before a final review by the authors.

#### Multidimensional Self-Esteem Scale

The AF5 scale (García and Musitu, 1999) assesses self-esteem in five domains: academic, social, emotional, family, and physical. Each domain is measured by six items with scores ranging from 0.1 to 9.99. The AF5 was originally developed and validated in Spain with a sample of 6,500 subjects (García and Musitu, 1999). The factor structure of the instrument was confirmed both with exploratory (García and Musitu, 1999) and CFAs (Tomás and Oliver, 2004; García et al., 2011) and no method effect appears to be associated with negatively worded items (Tomás and Oliver, 2004; García et al., 2011). The AF5 has been properly validated in the Basque (Elosua and Muñiz, 2010) and Catalan languages (Cerrato et al., 2011) and recently in English (García et al., 2013). This scale has been used in a large number of studies to consistently relate self-esteem to other variables (e.g., Fuentes et al., 2011). Finally, in previous studies, the ESPA29 parenting acceptance/involvement dimension has been related to higher child self-esteem, and the strictness/imposition dimension has been related to lower child self-esteem (Fuentes et al., 2011; García and Gracia, 2014).

### Statistical Analyses

The data was split randomly into two halves. On one of the two halves, a principal components analysis (PCA) with varimax rotation was carried out on the mothers’ and fathers’ scores of socialization practices. By extracting the maximum variance from the data set with each component, PCA provides an empirical summary of the data (Tabachnick and Fidell, 2013). PCA with varimax rotation is most commonly used as the initial stage of structural analysis and was the chosen method of analysis in the development of ESPA29 measure (López-Jáuregui and Oliden, 2009; Garaigordobil and Aliri, 2012).

In order to confirm the factorial structure obtained by the EFA, a CFA was carried out with Structural Equation Modeling Software (EQS) program using the second half of the data. The CFA technique allows the degree of adjustment of the model by the value of chi-squared to be obtained. However, chi-squared has serious problems of sensitivity to sample size (e.g., Bentler and Bonett, 1980; Cheung and Rensvold, 2002; García et al., 2006). Therefore, other fit indexes have been developed which have the advantage of pre-established cut-off criteria (e.g., Cheung and Rensvold, 2002; García et al., 2008, 2011; Murgui et al., 2012). We applied the following indexes: χ^2^/gl, a score of 2.00–3.00 or lower is indicative of a good fit; root mean squared error of approximation (RMSEA), values lower than 0.08 are considered acceptable; normed fit index and comparative fit index, NFI and CFI, whose value must exceed 0.90; and the information criterion of Akaike, AIC (Akaike information criterion), where the lowest value indicates the highest parsimony (Akaike, 1987). The estimation method was the maximum likelihood (ML), which, although assuming multivariate normality, is reasonably robust to its non-compliance (Curran et al., 1996). The criteria used are in line with those proposed by Hu and Bentler (1999) and are the usual utilized in this type of analysis (Martínez P. et al., 2012). Once the structure was verified separately for the practices of the mother and for the practices of the father, a multigroup analysis was carried out according to gender, using the usual procedure in these cases (Murgui and Musitu, 2011). First, the unconstrained model is calculated without any restrictions across parameters, and then, a new constrained model is calculated. If the difference in chi-squared values between the unconstrained model and the constrained model remains non-significant, it can be concluded that there is invariance between boys and girls, so the values of the restricted parameters are equivalent in both sexes. Moreover, the ESPA29 scale’s dimensions were related to self-esteem, which was measured through five dimensions with the AF5 instrument (García and Musitu, 1999), using Pearson correlation.

## Results

### Exploratory Factor Analysis

With one of the two halves of the data (456 participants), an EFA with Kaiser criterion and varimax rotation was carried out on the scores of the socialization practices of the ESPA29. The Kaiser-Meyer-Olkin (KMO) value was 0.62 for the father’ practices and 0.60 for the mother’ practices. The Bartlett test was significant for the fathers’ (χ^2^_21_ = 812.38; *p* < 0.01) and the mothers’ practices (χ^2^_21_ = 741.52; *p* < 0.01). The factor solution of the fathers’ scores explained 62.16% of the total variance, with two factors with eigenvalue equal to or greater than 1.0. Factor I (acceptance/involvement) explained 33.56% and Factor II (strictness/imposition) explained 28.60%. In the same way, the factor solution of the mothers’ scores explained 58.39% of the total variance, Factor I 31.46% and Factor II 26.93%. In both cases, the fathers’ and the mothers’ scores, the acceptance/involvement factor was made up of the warmth and reasoning subscales, loading positively onto the factor, whereas the indifference and detachment subscales loaded negatively. The factor loadings of the subscales in this factor ranged between 0.70 and 0.84 in the practices of the father and between 0.60 and 0.83 in the practices of the mother. In both, the fathers’ and the mothers’ scores the strictness/imposition factor was made up of the subscales of revoking privileges, verbal scolding, and physical punishment. These subscales loaded positively between 0.64 and 0.88 in fathers’ scores and between 0.58 and 0.87 in the mothers’ scores. Factor loadings of the subscales for both parents are shown in **Table 1**.

**Table 1:** Principal components analysis with two factors and varimax rotation of fathers’ and mothers’ parenting practices.

	Father		Mother	
		
	A/I	S/I	A/I	S/I
Warmth (He/she shows warmth)	0.84	0.12	0.83	-0.09
Indifference (He/she seems indifferent)	-0.76	0.28	-0.77	0.26
Detachment (It’s the same to him/her)	-0.70	-0.09	-0.60	0.06
Reasoning (He/she talks to me)	0.74	-0.11	0.72	0.20
Verbal scolding (He/she scolds me)	0.02	0.85	-0.04	0.82
Physical punishment (He/she hits me)	0.14	0.64	-0.19	0.58
Revoking privileges (He/she takes something away from me)	-0.12	0.88	0.11	0.87
% Variance	33.56	28.60	31.46	26.93


*A/I, acceptance/involvement; S/I, strictness/imposition.*

### Confirmatory Factor Analysis

A CFA was carried out on the second half of the data (455 participants). Given the high value of Mardia’s coefficient (36.00 for the fathers’ and 74.74 for the mothers’ scores), robust indicators were utilized. The fit of the models was not appropriate (**Table 2**, models Father 1 and Mother 1), thus we examined the indexes of modification and set the covariation restrictions free. Hence, the covariation between the following variables was included (fathers and mothers, respectively): detachment and revoking privileges (*r* = -0.26; *r* = -0.15), detachment and verbal scolding (*r* = -0.46; *r* = -0.44), reasoning and indifference (*r* = -0.67; *r* = -0.66), reasoning and verbal scolding (*r* = 0.72; *r* = 0.74), reasoning and revoking privileges (*r* = 0.68; *r* = 0.60). All the correlations were statistical significant (α < 0.01). Moreover, the correlation between the acceptance/involvement and strictness/imposition of both, the father (*r* = 0.29, *p* < 0.01) and the mother (*r* = 0.31, *p* < 0.01) was introduced. With these modifications, both CFA’s showed acceptable values (**Table 2**, models Father 2 and Mother 2).

**Table 2:** Confirmatory factor analysis of fathers’ and mothers’ parenting practices.

Model	S–Bχ^2^	*df*	S–Bχ^2^/*df*	CFI	IFI	NFI	AIC	RMSEA (90% CI)
Father 1	172.71**	14	12.34	0.78	0.78	0.77	144.71	0.151 (0.131–0.171)
Mother 1	177.54**	14	12.68	0.77	0.77	0.76	149.53	0.131 (0.14–0.148)
Father 2	26.26**	8	3.28	0.98	0.98	0.97	10.26	0.068 (0.040–0.097)
Mother 2	24.66**	8	3.08	0.98	0.98	0.97	8.66	0.055 (0.031–0.081)
Father 2U	58.16**	16	3.64	0.95	0.95	0.94	26.16	0.065 (0.048–0.084)
Mother 2U	34.72**	16	2.17	0.97	0.98	0.95	2.72	0.042 (0.022–0.061)
Father 2R	69.28**	22	3.15	0.98	0.97	0.94	7.48	0.047 (0.030–0.063)
Mother 2R	41.58**	22	1.89	0.97	0.97	0.95	-2.42	0.036 (0.019–0.053)


*S–Bχ^*2*^, Satorra–Bentler chi-squared; *df*, degrees of freedom; RMSEA, root mean squared error of approximation; IFI, incremental fit index; NFI, normed fit index; CFI, comparative fit index; AIC, Akaike information criterion. All indexes are the robust version; U, multigroup unrestricted model; R, multigroup restricted model. In model 2, covariation between variables and dimensions was added. ^∗∗^*p* < 0.01.*

The factor loadings of parental practices (**Table 3**) and the correlations between parenting practices are consistent with the theoretical approach. In addition, the factor loadings and the final structure replicated those obtained in the EFA. The correlation between the two dimensions presented values less than 7% of the shared dimensions variance.

**Table 3:** CFA standardized factor loadings of fathers’ and mothers’ parenting practices.

	Father		Mother	
		
	A/I	S/I	A/I	S/I
Warmth (He/she shows warmth)	0.67**	-	0.65**	-
Indifference (He/she seems indifferent)	-0.96^a^	-	-0.92^a^	-
Detachment (It’s the same to him/her)	-0.43**	-	-0.37**	-
Reasoning (He/she talks to me)	0.75**	-	0.70**	-
Verbal scolding (He/she scolds me)	-	0.87^a^	-	0.87^a^
Physical punishment (He/she hits me)	-	0.43**	-	0.33**
Revoking privileges (He/she takes something away from me)	-	0.73**	-	0.64**


*A/I, acceptance/involvement; S/I, strictness/imposition. ^a^Fixed to 1 during estimation. ^∗∗^*p* < 0.01.*

For the parenting practices of the mother and the father, the multigroup analysis was performed. First, the unrestricted multigroup model was calculated (Father 2U model and Mother 2U model). The models calculated for both parenting practices of the father and of the mother showed a good multi-sample adjustment, suggesting a common factor structure across the two genders.

Then, in each model, the paths of the practices in their dimension and the covariation between the two dimensions were fixed. This restricted model (Father 2R and Mother 2R model) did not imply, in comparison with the unrestricted model, a significant increase in the value adjustment of χ^2^, nor in the practices of the father ($\chi_{6}^{2}$ = 11.12, *p* > 0.05), nor in the case of the practices of the mother ($\chi_{6}^{2}$ = 6.86, *p* > 0.05). Thus, the factor loadings in both dimensions and the correlation between acceptance/involvement and strictness/imposition are equivalent between both sexes, and for the fathers’ and mothers’ scales.

### Descriptive Statistics and Internal Consistency

The classic descriptive indexes for each scale and subscale of the ESPA29, arithmetical means and standard deviation values, are shown in **Table 4**. The alpha coefficient of the acceptance/involvement dimension was 0.96. The alpha coefficient for the mothers’ scores in this dimension was 0.98, and was also 0.98 for the fathers’ scores in this dimension. The strictness/imposition dimension had a coefficient value of 0.98. For the mothers’ scores in this dimension, the alpha was 0.98, and was also 0.98 for the fathers’ scores. With respect to the individual subscales, the alpha coefficients were as follows: warmth, 0.90 for the mothers’ behavior and 0.89 for the fathers’; indifference, 0.90 for mothers and 0.89 for fathers; reasoning, 0.90 for mothers and 0.89 for fathers; detachment, 0.90 for mothers and 0.89 for fathers; verbal scolding, 0.91 for mothers and 0.89 for fathers; physical punishment, 0.90 for mothers and 0.89 for fathers; and revoking privileges subscale had alpha values of 0.90 for mothers and 0.89 for fathers. Finally, the Cronbach’s alpha of the total 212-item scale was 0.99. The alpha value for the 116 items for mothers was 0.99, and for the 116 items for fathers was also 0.99. Those alpha coefficients with the total scale were calculated in order to check that there is no malfunctioning or internal consistency problem with the items or with the scales, since all the items are measuring parts of the same construct, which is parental socialization.

**Table 4:** ESPA29 descriptive indexes.

	Mother				Father			
		
	*M*	*SD*	Min	Max	*M*	*SD*	Min	Max
Acceptance/involvement	2.97	0.52	1.48	4.00	2.79	0.57	1.03	4.00
Strictness/imposition	1.53	0.41	1.00	3.58	1.48	0.38	1.00	3.08
Warmth	2.56	0.83	1.00	4.00	2.34	0.82	1.00	4.00
Reasoning	2.70	0.72	1.00	4.00	2.54	0.72	1.00	4.00
Indifference	1.95	0.84	1.00	4.00	2.14	0.89	1.00	4.00
Detachment	1.43	0.45	1.00	3.44	1.56	0.53	1.00	4.00
Revoking privileges	1.54	0.55	1.00	3.94	1.49	0.51	1.00	3.63
Verbal scolding	1.99	0.75	1.00	4.00	1.90	0.69	1.00	3.88
Physical punishment	1.06	0.22	1.00	3.63	1.05	0.18	1.00	2.69


### Relation to Self-Esteem

The acceptance/involvement dimension of the ESPA29 scale related positively to academic, social, family, and physical self-esteem, whereas the strictness/imposition dimension of the scale was related negatively with academic, social, emotional, and family self-esteem (**Table 5**). The effect size of the correlations is similar to those reported in other studies that analyze the relation between parenting and self-esteem (Felson and Zielinski, 1989; Barber et al., 1992).

**Table 5:** Correlations and *R*^2^ between two major parental socialization dimensions with five self-esteem dimensions.

Self-esteem		Acceptance/involvement		Strictness/imposition	
			
	*M* (*SD*)	*r*	*R*^2^	*r*	*R*^2^
Academic	7.58 (1.90)	0.226^∗∗^	0.051	-0.089^∗∗^	0.008
Social	7.60 (1.49)	0.207^∗∗^	0.043	-0.087^∗^	0.008
Emotional	6.28 (1.95)	-0.053	0.003	-0.074^∗^	0.005
Family	7.43 (2.09)	0.534^∗∗^	0.285	-0.357^∗∗^	0.127
Physical	7.18 (1.89)	0.234^∗∗^	0.055	-0.033	0.001


*^∗^*p* < 0.05, ^∗∗^*p* < 0.01.*

## Discussion

Overall, the results of this work validate the English version of the ESPA29 Parental Socialization Scale. The theoretical two factor structure of the Parental Socialization Scale ESPA29 is clearly identified by both EFA and CFA in the United States data. The results of the PCA show that the subscales of warmth and reasoning of both mothers’ and fathers’ behavior loaded positively onto the acceptance/involvement dimension. Additionally, the subscales of indifference and detachment loaded negatively onto this dimension for both parents’ scores. Furthermore, the remaining three subscales—physical punishment, verbal scolding, and revoking privileges—all loaded positively onto the strictness/imposition dimension in the case of both parents’ behavior.

The CFA fully corroborates the theoretical structure of the Parental Socialization Scale ESPA29, supporting to the two dimensions of parental conduct proposed in the ESPA29. The CFA replicated the two-factor structure with appropriate fit indexes. The two axis dimensions reflect two main persistent patterns of parental conduct (Steinberg, 2005), which being orthogonal (the two are not related and behavior in one does not predict behavior in the other), must be analyzed together in order to determine the style of socialization that characterizes parental behavior toward the child (Grusec and Lytton, 1988; Darling and Steinberg, 1993; Steinberg, 2005). Unlike previous studies with the ESPA29 scale that only applied EFA with Procrustes Rotation (Marchetti, 1997; Musitu and García, 2001; Martínez and García, 2008; López-Jáuregui and Oliden, 2009; Martínez et al., 2011, 2013; Martínez I. et al., 2012; García and Gracia, 2014; García et al., 2015) the present study has applied the CFA. Furthermore, we have contrasted the gender invariance of factor loadings for fathers’ and mothers’ practices with the multigroup factor confirmatory analysis. These results are fully consistent with those obtained in the normalization of the original scale (Musitu and García, 2001) and with those from previous adaptations into other languages, reinforcing the universality of the practices measured by the Parental Socialization Scale ESPA29 (López-Jáuregui and Oliden, 2009; Grusec, 2012; Martínez I. et al., 2012). The results demonstrate that the ESPA29’s structure and conceptualization are the same among both fathers and mothers (Maccoby and Martin, 1983; Lamborn et al., 1991; Steinberg et al., 1994; Musitu and García, 2001).

Therefore the existence of two independent dimensions of parental conduct in the process of family socialization is supported (Maccoby and Martin, 1983; Darling and Steinberg, 1993). This operationalization of parenting is congruent with that of a large number of instruments used to analyze parental conduct. As Steinberg (2005) highlights, the majority of studies on parenting styles has operationalized one of the dimensions using measures of parental warmth and acceptance while the other has been based on parental firmness. Thereby, the dimensions of strictness/imposition and acceptance/involvement (Steinberg et al., 1994), acceptance/rejection and control, or the dimensions of acceptance/involvement and strictness/imposition as they are named in the ESPA29 (Rohner, 1990; Musitu and García, 2004), have been used.

Furthermore, the multigroup analysis shows that the structure of the scale is equivalent for adolescent males and females, in both mothers’ and fathers’ scores. The subscales of warmth and reasoning of both mothers’ and fathers’ behavior loaded positively onto the acceptance/involvement dimension and the subscales of indifference and detachment loaded negatively onto the strictness/imposition dimension. The subscales of physical punishment verbal scolding, and revoking privileges loaded positively onto the strictness/imposition dimension. Adolescent males and females show equivalent loadings in the paths of each subscale of the two dimensions, acceptance/involvement and strictness/imposition.

Additionally, the parenting practices of the scale are related to one of the most widely utilized adolescent adjustment criteria variables: self-esteem (Felson and Zielinski, 1989; Barber, 1990; Musitu and García, 2001; López-Jáuregui and Oliden, 2009; Fuentes et al., 2011) in order to have an external validity index. The results show that the acceptance/involvement dimension, which includes the use of practices of reasoning and warmth, is positively related with self-esteem, whereas the strictness/imposition dimension, which includes the use of the verbal scolding, physical punishment and revoking privileges practices, in negatively related with adolescents self-esteem. These results are similar to those reported in other studies that analyze the relation between parenting and self-esteem (Barry et al., 2008), showing that positive parenting tends to be associated with high self-esteem, whereas negative parenting is associated with low self-esteem (Lamborn et al., 1991; Steinberg et al., 1994; Calafat et al., 2014). More specifically, other studies using the ESPA29 have reported similar results (Fuentes et al., 2011; García and Gracia, 2014). Although this is a first approximation of the relation of the practices of the ESPA29 with a criterion variable in a United States sample, future research should analyze the relation between parenting styles assessed with the ESPA29 in United States samples and other criteria variables that reflect personal and social adolescent adjustment. In the same way, other analyses, such as testing the concurrent validity of the ESPA29 with a United States sample, should be contemplated in the future. Finally, it would be advisable that the analysis of this study be extended to other age ranges and that specifically CFA be carried out with samples from different countries. Nevertheless, the results of this study show that the English version of the ESPA29 is adequate for measuring parental socialization in English-speaking adolescents.

## Author Contributions

IM, EC, OG, and SM had participated in the intellectual content, the analysis of data, and the writing of the work. IM, EC, OG, and SM had reviewed the final version of the work and they approve it for publication.

## Conflict of Interest Statement

The authors declare that the research was conducted in the absence of any commercial or financial relationships that could be construed as a potential conflict of interest. The reviewer AP and handling Editor declared their shared affiliation, and the handling Editor states that the process nevertheless met the standards of a fair and objective review.
